# Always look on the bright side of life: Individual differences in visual attentional breadth for understanding temperament and emotion regulation in adolescents

**DOI:** 10.3389/fpsyg.2023.1094108

**Published:** 2023-03-02

**Authors:** Marie-Lotte Van Beveren, Jolien Braet, Rudi De Raedt, Maud Grol, Laura Wante, Caroline Braet

**Affiliations:** ^1^Clinical Developmental Psychology, Department of Developmental, Personality and Social Psychology, Ghent University, Ghent, Belgium; ^2^Department of Experimental Clinical and Health Psychology, Ghent University, Ghent, Belgium

**Keywords:** temperament, adolescence, emotion regulation, positive emotionality, attention

## Abstract

**Introduction:**

Cognitive-affective models of depression show that negative and positive emotionality differentially confer risk for depression through maladaptive and adaptive emotion regulation (ER) strategies respectively. Yet, no research has examined the mechanisms through which these temperament traits shape individual differences in ER. The current study explored the mediating role of attentional breadth for emotional information in the distinct pathways from temperament to ER strategies in adolescents.

**Methods:**

The hypotheses were tested in a selected sample of 71 adolescents (*M* = 14.15, *SD* = 1.90; 62% girls) using a previously validated measure of visuospatial attentional breadth.

**Results:**

First, positive emotionality was positively associated with attentional breadth for positive stimuli and temperamental vulnerable adolescents showed deficits in the processing of positive stimuli when presented far from the center of the visual field. Second, attentional breadth towards neutral stimuli was positively related to adaptive ER strategies. Third, no evidence was found for the proposed mediation models. However, post-hoc analyses provided preliminary evidence for a reversed mediation model in which adaptive ER strategies mediate the relationship between temperament and attentional breadth towards neutral stimuli.

**Discussion:**

The results underscore the apparent complexity of the relations between temperament, attentional breadth, and ER and point out the need for further research in order to inform early intervention.

## 1. Introduction

Major depressive disorder (MDD) is a debilitating affective disorder that increases markedly in adolescence, with approximately 10.5% of European adolescents meeting the criteria for clinical depression and more than 29% reporting subthreshold depressive symptoms (Balazs, 2013; Shorey et al., 2022). Given the number of undesirable outcomes associated with early-onset depression (Burcusa and Iacono, 2007; Nock et al., 2013), research into the factors influencing the development of depressive symptoms during this critical formative period is needed for the development of effective prevention and intervention programs targeting early-onset depression.

Depression is a disorder characterized by an excess of negative emotion (sad mood) and the inability to experience positive affect or pleasure (anhedonia; American Psychological Association, 2013). Therefore, clinical developmental researchers have posited that both the temperamental traits of high negative emotionality and low positive emotionality are associated with the onset and maintenance of depression in childhood and adolescence (Clark and Watson, 1991; Lonigan et al., 2003; Bould et al., 2014). Integrated cognitive-affective models of depression (e.g., Yap et al., 2007; Hyde et al., 2008) have suggested that the effects of temperament on depression may be mediated by the use of emotion regulation strategies, with maladaptive emotion regulation strategies mediating the specific effect of high trait negative emotionality on depression and adaptive emotion regulation strategies mediating the unique effect of low trait positive emotionality on depression. Although research (Verstraeten et al., 2009, 2012; Mezulis et al., 2011; Yap et al., 2011; Mezulis and Rudolph, 2012; Harding et al., 2014; Hudson et al., 2015; Van Beveren et al., 2017, 2019a) supports the theoretical premises posited by such integrated cognitive-affective models, no research has examined the mechanisms through which temperament traits shape individual differences in emotion regulation strategies.

### 1.1. Literature review

Clinical developmental research indicates that temperament traits, i.e., the biologically-based individual differences in emotional and behavioral reactivity that appear early in life and are stable across time and situations (Rothbart and Bates, 2006), can confer vulnerability for psychopathology in general and depression specifically (Muris, 2006; Muris et al., 2007). Temperamental theories typically differentiate between trait negative emotionality (NE) and trait positive emotionality (PE) as two temperament traits that predispose individuals to experience different levels of emotions (both valence and intensity) as well as to be more or less reactive to emotions (Rothbart et al., 2000). Trait NE specifically refers to one’s susceptibility to negative emotions such as depressed mood and anxiety (Rothbart et al., 2000), and vulnerability for psychopathology including depression (Lonigan et al., 2003). Trait PE refers to one’s reactivity to positive emotions such as cheerfulness and states of positive engagement (Rothbart et al., 2000), and resilience to psychological problems. Moreover, an extensive corpus of research has consistently shown that high trait NE serves as a *general*, non-specific, risk factor for psychopathology (Verstraeten et al., 2009; Wetter and Hankin, 2009; Mezulis and Rudolph, 2012; Tackett et al., 2013; Bould et al., 2014; Dolcini-Catania et al., 2020), whereas low trait PE serves as a specific risk factor for depression (Clark and Watson, 1991; Lonigan et al., 2003). These patterns have been found both concurrently and prospectively in children, adolescents, and adults alike (De Pauw and Mervielde, 2010; Klein et al., 2011; Dolcini-Catania et al., 2020).

Temperamental theories and related empirical research state that NE and PE do not exist in isolation but instead jointly predict vulnerability to depression (Dinovo and Vasey, 2011; Vasey et al., 2013, 2014; Van Beveren et al., 2019b). For example, research using a variable-centered (Van Beveren et al., 2019b) and person-centered (Van Beveren et al., 2020) data-analytical approach indicates that a temperamental constellation characterized by both *high* trait NE and *low* trait PE particularly reflects temperamental vulnerability to developing depressive symptoms in youth, while a combination of *low* trait NE and *high* trait PE is considered a temperamental resilient profile that buffers youth against the storm-and-stress that characterizes the adolescent phase. Yet, given that negative and positive emotions have distinct cognitive, behavioral, and psychophysiological effects (Fredrickson, 2001; Garland et al., 2010), it is unlikely that similar mechanisms underly the developmental pathways from temperamental vulnerability and resilience to depression. Research disentangling both pathways is needed in order to provide clear insights into the mechanisms through which temperament traits confer differential vulnerability to depression. Given that temperament traits are difficult to change because of their strong genetic and neurobiological underpinnings (Rothbart and Posner, 2015), such a research approach could inform early intervention and prevention programs about which influenceable mechanisms to target in order to diminish the harmful consequences associated with one’s temperamental vulnerability.

Recently, researchers have started to integrate insights from temperament theory (Clark and Watson, 1991; Lonigan et al., 2003) and well-examined cognitive-behavioral vulnerability models of depression, such as the renowned response style theory (Nolen-Hoeksema, 1991; Nolen-Hoeksema and Girgus, 1994), for understanding the development and maintenance of depressive symptoms in adolescence. Such integrated cognitive-affective models of depression (e.g., Yap et al., 2007; Hyde et al., 2008) suggest that individual differences in emotion regulation develop in accordance with one’s temperament to the extent that adolescents manage their emotions in a way that is consistent with their temperamental-based tolerances (Thompson, 2006; Hyde et al., 2008). According to these models, emotion regulation thus functions as one possible mechanism through which temperament traits increase vulnerability to depression. Emotion regulation (ER) refers to a range of processes through which individuals can change the nature, frequency, and duration of emotions (Gross, 1999). ER strategies refer to the ways in which individuals actively and goal-oriented regulate their emotions (Koole, 2010), and can be broadly divided into two categories based on their long-term effects on affect, behavior, cognition, and their overall association with psychopathology (Aldao et al., 2010; Schäfer et al., 2017). Some ER strategies are considered maladaptive (e.g., rumination, aggression, avoidance) because they have been associated with more overall maladjustment in the long-term (Aldao et al., 2010; Schäfer et al., 2017) whereas others have been labeled adaptive (e.g., reappraisal, positive refocusing, problem solving) as they have been related to more overall emotional wellbeing in the long-term. Integrated cognitive-affective models of depression (e.g., Yap et al., 2007; Hyde et al., 2008) posit that trait NE and trait PE each play a specific and distinct role in shaping individual differences in ER strategies with trait NE contributing to the development of maladaptive ER strategies and trait PE contributing to the progression of adaptive ER strategies. This theoretical premise is further substantiated with recent empirical evidence in young-adults (Harding et al., 2014; Hudson et al., 2015) and youth (Van Beveren et al., 2016a, 2017, 2019a; Liu et al., 2022) showing that high trait NE is particularly associated with the overall and daily use of maladaptive ER strategies such as rumination, whereas low trait PE has repeatedly and specifically been linked to an inadequate use of adaptive ER strategies such as positive refocusing.

To date no research has examined the mechanisms through which temperament traits shape individual differences in ER strategies. A useful framework to understand the differential effects of trait NE and PE for ER is the broaden-and-build theory of positive emotions (Fredrickson, 1998, 2001). According to this theory, negative and positive emotions have distinct – but complementary – cognitive, behavioral, and psychophysiological effects. First, it is theorized that negative emotions restrict people’s thought-action repertoire and action tendencies (e.g., fight in anger or flight in fear, but see Jäger and Rüsseler, 2016) which leads to attentional narrowing and consequently to repetitive thoughts or stringent behavior. Although such narrowed thought-action repertoires are adaptive within a life-threatening situation requiring quick and decisive action that carries direct and immediate benefit (i.e., save our lives), they may become maladaptive in the long-run and even contribute to overall vulnerability to psychopathology over time (De Raedt and Koster, 2010; Gotlib and Joormann, 2010). So, whereas acute and short-term reactions to a stressor are considered adaptive for our survivability, chronic psychophysiological reactivity toward stress and related narrowed action and thinking patterns are maladaptive and detrimental for our emotional wellbeing in the long-run. Positive emotions, however, are theorized to have a complementary effect: positive emotions are thought to broaden people’s cognitive abilities and behavioral repertoires which encourage them to be open to new experiences and discover novel lines of thought or action, and contribute to building personal resources over time (Fredrickson, 1998, 2001; Jäger and Rüsseler, 2016). Hence, the broadened thought-action repertoires resulting from positive emotions are likely to be adaptive at the long-term. Interestingly, the broaden-and-build theory proposes that the broadening effects of positive emotions may constitute an underlying mechanism in the reciprocal relation between positive emotions and resilience (Fredrickson and Joiner, 2002). This suggests that especially individuals with high trait PE and low trait NE (i.e., resilient adolescents) will show more overall benefits from experiencing positive emotions in their daily lives in the form of attentional broadening, whereas adolescents who carry temperamental vulnerability – as reflected by high trait NE and low trait PE – may show less broadening due to their generally reduced reactivity toward positive emotions. Instead, their narrowed attentional scope and stringent behavioral and cognitive repertoire following from experiencing more negative emotions in general, may hamper them from escaping a negative spiral, which puts them at heightened risk of developing depression (Carl et al., 2013).

The differential effects of negative and positive emotions on people’s thought-action repertoire and the processing of emotional information have been investigated in adults using a plethora of different conceptualizations (e.g., global–local visual processing) mostly targeting higher level cognition (Isen and Daubman, 1984; Isen et al., 1987; Baas et al., 2008; Garland et al., 2010). Others investigated the differential effects of negative and positive emotions on *visual attentional breadth,* i.e., attentional scope (Grol and De Raedt, 2014). The results of these studies generally support the theoretical premises put forward by Fredrickson (2001) implying that, in adults, negative mood and depressive symptoms are associated with a more narrowed attentional scope (Derryberry and Reed, 1994; Basso et al., 1996; de Fockert and Cooper, 2014) whereas positive emotions are associated with a more broadened attentional scope (Derryberry and Reed, 1994; Gasper and Clore, 2002; Fredrickson and Branigan, 2005; Rowe et al., 2007). Yet, empirical evidence for this relationship remains equivocal as other studies failed to replicate the beneficial effects of positive emotions on visual attentional breadth (Bruyneel et al., 2013) or found this relationship to be dependent on depressive symptomatology (Grol and De Raedt, 2014). Furthermore, parallel research in younger age groups is practically non-existent and despite the call for studying how personal characteristics may influence attentional broadening (Grol and De Raedt, 2014; Grol et al., 2015), no study to date has investigated the effects of temperament on visual attentional breadth for emotional stimuli in adolescents.

**Figure 1:**
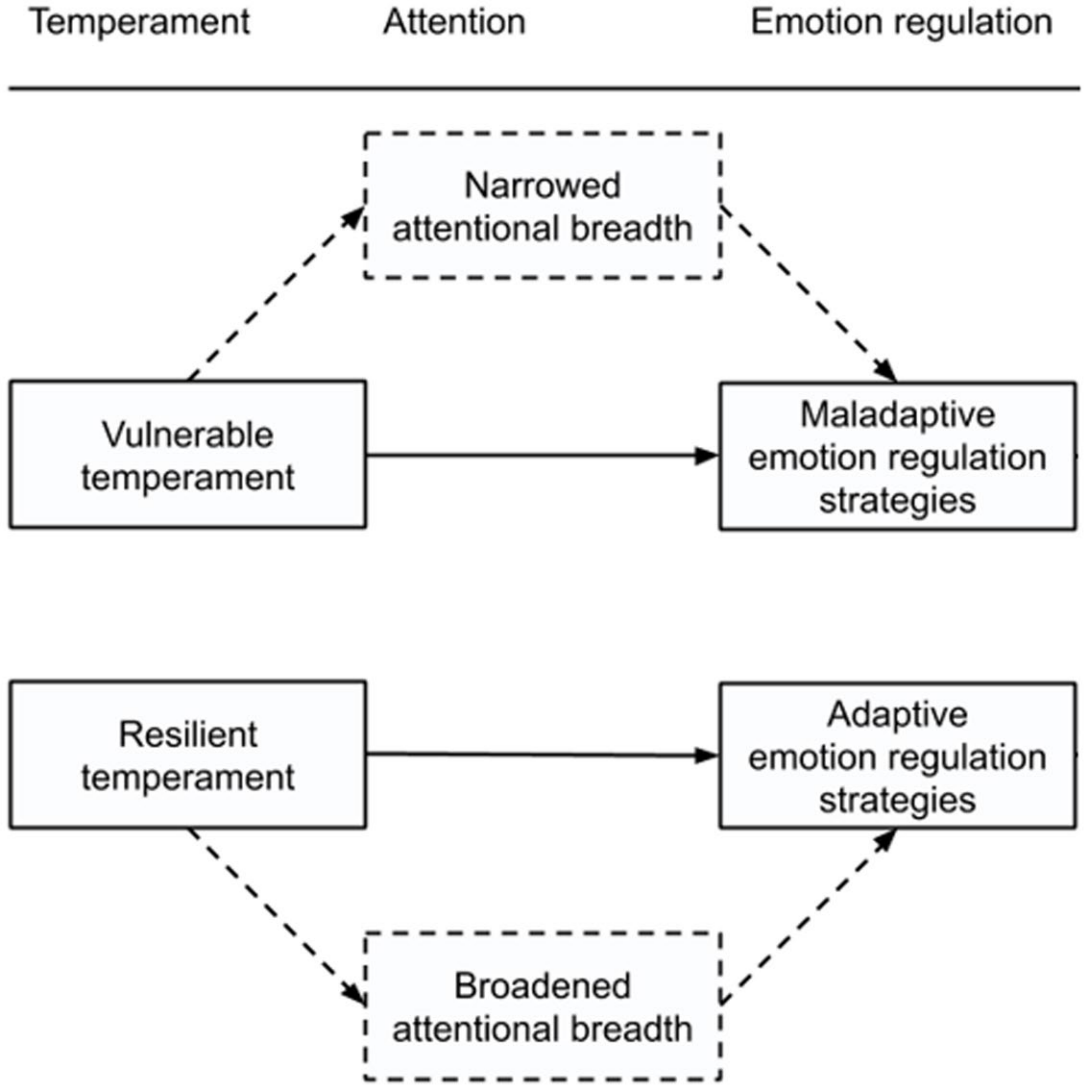
Proposed pathways through which temperament differentially shapes emotion regulation strategies. Hypotheses are indicated with dashed lines.

Attention is the starting point of information processing and initiates the ER process if indicated. More specific, attention is considered to be a crucial first step in the processing of emotional relevant information as it qualifies which information is encoded for further processing (Gross and Thompson, 2007) and thus determines the further course of the emotion generative and regulation process (Gross, 2013; Sanchez-Lopez et al., 2019). Theoretically it can be stated that temperamental vulnerable adolescents will naturally experience a heightened reactivity toward negative emotions from day-to-day, which results in more narrowed attention. The narrowed attentional scope, caused by negative mood, will limit the activated thought-action repertoires of the temperamental vulnerable adolescent. In turn, these narrowed through-action repertoires increase the likelihood that thoughts become repetitive, narrowed, and/or pessimistic and hamper adaptive ER efforts, such as putting things in perspective or effective problem-solving behavior, in the long-term. Instead, the narrowed attentional scope may increase the overall tendency to use maladaptive ER strategies such as rumination, i.e., repetitive thinking about one’s feelings and problems, as the default way of regulating one’s negative emotions (Nolen-Hoeksema et al., 2008). In short, it can thus be presumed that temperamental vulnerability enhances one’s general tendency to use maladaptive ER strategies such as rumination over time through its narrowing effect on the attentional scope (Figure 1). This theoretical assumption is supported by research on the attentional scope model of rumination (for review see Whitmer and Gotlib, 2013) suggesting that a more narrowed attentional scope is positively associated with the tendency to ruminate in response to distress. However, a previous experimental study on the attentional scope model in college students (Grol et al., 2015) also suggest that the reversed relationship may just be as likely, i.e., that trait maladaptive ER strategies at their turn also lead to a more narrowed attentional scope over time (Grol et al., 2015). Such a reversed relationship could additionally explain the self-perpetuating nature of maladaptive ER strategies.

On the other hand, temperamental resilient individuals usually experience heightened reactivity to positive affect, which could theoretically result in more broadened attention and beneficial effects on the default ER-strategy repertoire. Broadened attentional scope caused by positive mood widens the array of percepts, thoughts, and actions presently in mind and allows more flexible processing of a wider range of information (Fredrickson, 1998, 2001). For example, experimental evidence has documented that people experiencing positive affect show patterns of thought that are flexible and inclusive, creative, integrative, open to information, and efficient (Isen et al., 1991; Estrada et al., 1997; Phillips et al., 2002). Given that the common denominator of adaptive ER strategies is to pursue a wider range of thoughts (e.g., reappraisal, cognitive problem solving) and actions (e.g., support seeking, behavioral problem solving, expression) than currently in mind, a broadened visual attentional scope may increase the tendency to develop adaptive ER strategies over time (Aspinwall, 1998; Fredrickson and Joiner, 2002) because it enables the individual to consider a wider range of information that is available within the environment. This is further evidenced by research on the broaden-and-build theory (for review see Garland et al., 2010) suggesting that positive emotions facilitate coping with stress and adversity – which in turn predicts future experiences of positive emotions (Fredrickson and Joiner, 2002). Through broadening the attentional scope, temperamental resiliency is thus believed to enhance one’s general tendency to use adaptive ER strategies (Figure 1). However, given the complexity of the aforementioned relations and the fact that the relations among temperament traits, ER strategies, and visual attentional breadth in adolescents is still insufficiently addressed in previous work, more research is warranted.

Considering that research on attentional functioning related to emotional responding and regulation in adolescents is still in its infancy, the current study had three goals. First, we aimed to examine the relationship between temperament and visual attentional breadth for emotional (i.e., positive, neutral, and negative) stimuli with a previously validated performance-based measure of visuospatial attentional breadth (Bosmans et al., 2009; Grol and De Raedt, 2014; Grol et al., 2015). Second, we aimed to investigate the unique relationship between self-reported trait ER strategies (adaptive and maladaptive) and visual attentional breadth for emotional stimuli while considering temperamental differences. Lastly, we aimed to examine whether individual differences in visual attentional breadth for emotional stimuli mediate the relationship between temperament and individual differences in ER strategies by testing the model presented in Figure 1.

Drawing from the broaden-and-build theory of positive emotions and prior work (Basso et al., 1996; Fredrickson, 2001; Gasper and Clore, 2002; Rowe et al., 2007), we hypothesized that temperamental vulnerable adolescents who are high in trait NE and low in trait PE would show a more narrowed attentional focus, especially toward negative and positive emotional stimuli, compared to temperamental resilient adolescents low in trait NE and high in trait PE who are thought to show a more broadened attentional focus for such emotional stimuli (as compared to vulnerable adolescents) which may reflect one’s capability of considering a wider range of alternative information. Second, we expected (Fredrickson, 2001; Garland et al., 2010; Whitmer and Gotlib, 2013) a more narrowed attentional focus for negative emotional stimuli to be positively related to a greater use of maladaptive ER strategies, whereas a more broadened attentional focus for positive emotional stimuli would indicate a greater reliance on adaptive ER strategies while taking into account temperamental differences. Finally, we expected the relation between temperament and ER strategies to be mediated by visual attentional breadth for emotional stimuli. More specific, we hypothesized the relationship between temperamental vulnerability and maladaptive ER strategies to be mediated by attentional narrowing toward negative emotional stimuli and the temperamental resilience – adaptive ER strategies relationship to be mediated by attentional broadening toward positive emotional stimuli.

## 2. Methods

### 2.1. Participants

Seventy-one adolescents aged between 11 and 18 years (*M* = 14.15, SD = 1.90; 62% girls) participated in the current study. Participants were selected from a screening sample of youth who participated in a larger, school-based study (conducted in several cities in Flanders, Belgium). All children assented to take part in follow-up studies, consent from their parents was also received. For the current study, we aimed to select individuals from the screening sample who scored high in trait NE, and low in PE (vulnerable), as well as individuals who scored low in trait NE, and high in PE (resilient; Clark and Watson, 1991; Vasey et al., 2014; Van Beveren et al., 2019b). Individuals were assigned to the vulnerable group if they scored high (≥ 38) on the negative emotionality (PANAS-NE) scale and low (≤ 43) on the positive emotionality (PANAS-PE) scale of the Positive Affect and Negative Affect Schedule for Children (PANAS-C; Laurent et al., 1999), whereas the resilient adolescents scored low (≤ 28) on the PANAS-NE scale and high (≥ 50) on the PANAS-PE scale. These scores refer to the 30^th^ (low scores) and 70^th^ (high scores) percentile PANAS-C scores from a large community-based sample of Flemish adolescents (see Van Beveren et al., 2017) consisting of 1,646 adolescents ranging from 7 to 16 years (*M* = 11.41, SD = 1.88; 54% girls). All participants filled out the PANAS-C, a measure on ER and a measure on depressive symptoms. Note that the cut-off scores are higher than previously established preliminary PANAS-C cut-off scores (PANAS-NE = 35; PANAS-PE = 36) in schoolchildren (Laurent et al., 2011). The research protocol of the current study was approved (ref: 2016/106/Marie-Lotte Van Beveren) by the Ethical Committee of the Faculty of Psychology and Educational Sciences at Ghent University (Belgium). Prior to the start of the current study, all participant signed informed assent, and their parents signed an informed consent form. After completing the study, participants were compensated with two cinema tickets.

### 2.2. Procedure

All participants were invited to the lab at the Faculty of Psychology and Educational Sciences of Ghent University (Belgium). One week beforehand, all adolescents completed self-report questionnaires on a secure online platform at home. On the day of testing, participants first explored the room to familiarize themselves with the lab-setting. During this time, the investigator guided the parent (s) to a waiting room. Parents were asked to fill out questionnaires on an iPad while waiting for their son or daughter. After informed consent/assent was provided, adolescents were asked to sit in front of a computer. A subsample of adolescents (*n* = 55; mean age = 13.69 years; 60% girls) first completed an affective Posner paradigm as described by Van Beveren et al. (2019c) followed by a short break. After watching a 3–min clip from the film Alaska’s Wild Denali (Hardesty, 1997) to guarantee a neutral baseline for all participants, adolescents completed the attentional breadth task (for methods see below). After the task, adolescents completed a series of additional assignments (which are irrelevant for the current study): (1) a questionnaire was filled in which assessed physical activity, (2) the block design and vocabulary subtests of the WISC-IV (Wechsler, 2003) were conducted, (3) the SCID-Junior interview (Wante et al., 2021) to assess DSM-5 symptoms (American Psychological Association, 2013) was completed, and (4) finally all adolescents were weighed and measured.

### 2.3. Experimental task

**Figure 2:**
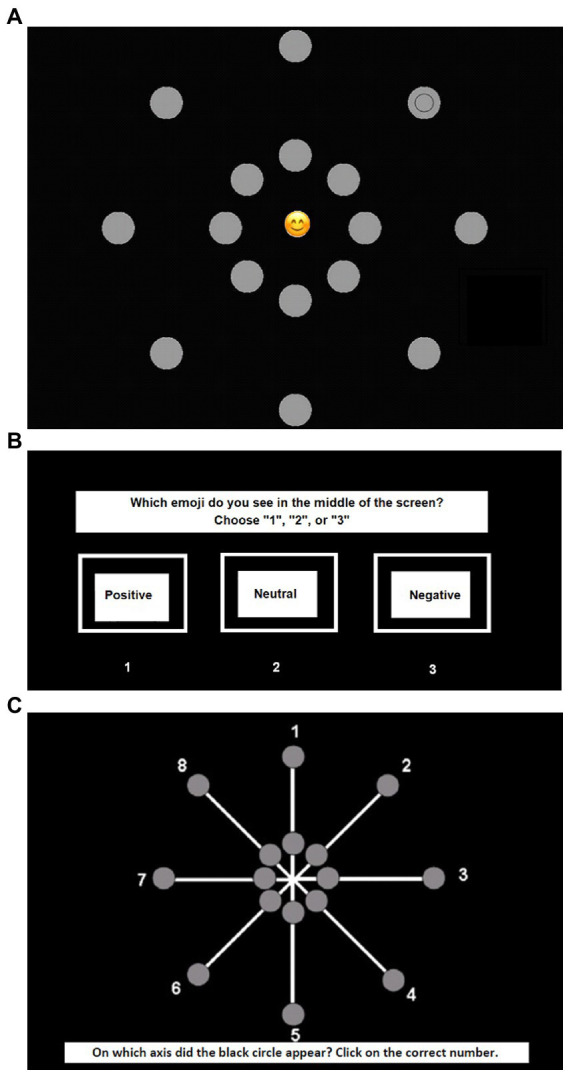
Stimulus presentation of the attentional breadth task. **(A)** The appearance of the central emoji and the target stimulus (black circle); **(B)** identification of the valence of the central emotion; **(C)** identification of the target stimulus position (black circle).

An adaptation of a task measuring attentional breadth in relation to centrally presented stimuli (Bosmans et al., 2009) was used in the current study to assess attentional broadening and narrowing. Participants were seated at a distance of 27 cm from a 19” CRT computer screen. A chin rest was used to ensure correct positioning. In each trial a picture of an Emoji (82 × 82 pixels) appeared in the center of the screen (Figure 2A). Emoji were taken from the Emoji Sentiment Ranking.^1^ For each condition (i.e., positive, neutral, and negative) eight Emoji were selected based on valence (negative = 1; positive = 5) and arousal ratings (low arousal = 1; high arousal = 5) obtained from prior validation (Vanden Berghe et al., 2020). The selected Emoji were negative (*M* arousal = 3.66), positive (*M* arousal = 4.40), and neutral (*M* arousal = 2.44).

Together with the presentation of the central Emoji, 16 dots with a diameter of 2 cm emerged around the Emoji in two concentric circles (Figure 2A). One circle appeared at 11.2 cm (i.e., far) from the central Emoji at 25° of the visual angle, the other circle appeared at 4.5 cm (i.e., close) from the central Emoji at 10° of the visual angle. The gray dots were arranged in pairs of two, one far and one close, situated on one of the eight invisible axes. A smaller black circle with a diameter of 1.3 cm appeared in one of the gray dots, either far or close. In order to prevent confounds of saccadic eye movements in search of the peripheral target (Ball et al., 1988), all stimuli were presented for 68 ms.

Participants were instructed to both correctly identify the valence of the corresponding emotion of the central Emoji (i.e., positive, neutral, and negative) and localize the black circle that appeared in one of the gray dots. Firstly, participants were asked to identify the valence of the emotion corresponding with the central stimulus (Figure 2B). There was no time limit to give a response. Secondly, participants were asked to identify the axis on which the target stimulus (i.e., the smaller black circle) had appeared (Figure 2C). Again, there was no time limit to give a response. The proportion of correctly localized target stimuli was used as the main dependent variable and was calculated based on trials in which participants also correctly identified the emotion corresponding with the central stimulus to make sure participants maintained attention to the center of the screen during the task.

The task consisted of eight practice trials with a presentation time of 250 ms, followed by eight practice trials with a presentation time of 68 ms similar to the test phase. The test phase consisted of 144 trials with six types of trials: positive close, positive far, neutral close, neutral far, negative close, and negative far which were randomly presented in three blocks consisting of 48 trials each.

### 2.4. Self-report questionnaires

#### 2.4.1. Temperament

During the screening phase all participants filled in the PANAS-C (PANAS-C; Laurent et al., 1999; see “Participants” section for more information). The PANAS-C is a self-report instrument that contains emotion items. Adolescents are asked to rate to which extend they usually experience each specific emotion on a 5-point Likert scale (1 = ‘very slightly’ to 5 = ‘very much’). The questionnaire consists of 30 items. All items can be divided in one of two subscales (i.e., NE and PE). The PANAS-C demonstrated good convergent and discriminant validity with child self-reports of depression and anxiety (Laurent et al., 1999). In the current study, Cronbach’s alphas for the NE and PE subscale were 0.83 and 0.88, respectively. Due to the sampling method, temperament was operationalized as a categorical variable named ‘group’ for which the values were (1) = vulnerable and (2) = resilient group.

#### 2.4.2. Emotion regulation strategies

The FEEL-KJ was used to assess ER strategies in children and adolescents aged 8 to 18 years (Grob and Smolenski, 2005; Braet et al., 2013). It consists of 90 items that measure different adaptive, maladaptive, and external ER strategies in response to three emotions, namely anger, fear, and sadness. Adolescents rate each item on a 5-point Likert scale (1 = ‘almost never’ to 5 = ‘almost always’). In the present study, the total adaptive (FEEL-KJ-AD; behavioral problem solving, cognitive problem solving, forgetting, acceptance, distraction, positive refocusing, and reappraisal) and total maladaptive (FEEL-KJ-MAL; giving up, aggression, rumination, self-devaluation, and withdrawal) subscales were calculated. Total scores on these scales comprise the separate scores on all three emotions and reflect general dispositions to cope with negative emotions. The FEEL-KJ has proven to be a valid and reliable questionnaire (Schmitt et al., 2012; Braet et al., 2013). Internal consistency for the FEEL-KJ-AD and the FEEL-KJ-MAL subscale in current study was 0.97, and 95, respectively.

#### 2.4.3. Affective states

Adolescents completed a paper-and-pencil rating of valence and arousal, the Self-Assessment Manikin (McManis et al., 2001) to provide self-report data on concurrent affective state during the lab visit. These ratings consisted of unlabeled line-drawings of manikins along a 9-point visual analog scale (VAS) ranging from 1 = ‘negative’ to 9 = ‘positive’ (valence) and 1 = ‘calm’ to 9 = ‘aroused’ (arousal). Adolescents provided a valence and arousal rating (T1) before and (T2) after the neutral baseline phase/before the attentional breadth paradigm and (T3) directly after the attentional breadth paradigm.

### 2.5. Data-analytic plan and task performance

For the analyses of the attentional breadth task, all trials were deleted in which the central Emoji was incorrectly identified, to make sure participants also maintained attention to the center of the screen during the task. The proportion of correctly identified targets on trials with correctly identified pictures served as the main dependent variable. Performance on the attentional breadth task was examined by performing a 3 Valence (Positive vs. Neutral vs. Negative) x 2 Distance (far vs. close) mixed ANOVA with the proportion of correctly localized peripheral targets as the dependent variable. To test our first two research questions, we ran two additional 3 Valence (Positive vs. Neutral vs. Negative) × 2 Distance (far vs. close) mixed ANOVAs: (1) one with group as a between subject-factor and (2) one with FEEL-KJ-AD and FEEL-KJ-MAL added as covariates (continuous factor).

In following the recommendations of Preacher and Hayes (2008) the proposed mediation models were tested using bootstrapping as provided by PROCESS (Hayes, 2013). Model 4 includes the option of testing a mediation model. Such a model suggests that the relationship between the independent variable (IV) and the dependent variable (DV) is partially or completely explained by the mediating variable(s) (M) included in the model. For mediation to occur, the *indirect* path between the IV and the DV through the M should be significant. In the current study, an accelerated-bias-corrected bootstrapping method with 5,000 estimates was used, to investigate whether the indirect effect was significantly different from zero (Preacher and Hayes, 2008).

For the mediating variables (M) and follow-up analyses pertaining to our first two research questions we calculated an Attentional Broadening Index (ABI) by subtracting the accuracy ‘target stimulus close to the central stimulus’ from the accuracy ‘target stimulus far from central stimulus’ for positive trials (posABI), neutral trials (neuABI), and negative trials (negABI) separately (Grol and De Raedt, 2014; Grol et al., 2015). Higher ABI scores imply higher attentional broadening.

We ran two mediation models (see dashed line Figure 1) in which multiple potential mediators (i.e., posABI, neuABI, and negABI) were tested (multiple mediation model): (1) the first model included group as the IV and FEEL-KJ-AD as the DV, (2) the second model included group as the IV and FEEL-KJ-MAL as the DV. Notably, in multiple mediation a specific indirect effect does not represent the ability of a given mediator M to mediate the effect of IV on DV. Rather, a specific indirect effect represents the ability of M to mediate the effect controlling for all other mediators. Thus, a specific indirect effect for M represents M’s unique ability to mediate the proposed relationship. This approach is preferred over testing several simple mediation models since the latter approach can lead to biased parameter estimates (Preacher and Hayes, 2008).

## 3. Results

### 3.1. Neutral video baseline

Results from paired sample *t*-tests revealed a slight but significant increase in valence (i.e., more positive) of affective state, *t*(62) = −2.42, *p* = 0.018, from T1 (*M* = 6.23, SD = 1.58) to T2 (*M* = 6.68, SD = 1.58), and a significant decrease in arousal, *t*(62) = 2.10, *p* = 0.040, from T1 (*M* = 3.61, SD = 1.88) to T2 (*M* = 3.23, SD = 1.68), indicating a successful neutral baseline phase. After completion of the task, we observed a significant decrease in the valence ratings of the experienced affect (*M* = 5.75, SD = 1.90; *t* (59) = −5.62, *p* < 0.001); and an increase in arousal (*M* = 4.65, SD = 2.08; *t* (59) = 3.72, *p* < 0.001). Importantly, there were no significant group differences in mood score (*t* (60) = −1.49, *p* = 0.142, *d* = 0.40) and arousal (*t* (60) = 1.38, *p* = 0.173, *d* = 0.37) after watching the Denali film clip (T2).

### 3.2. Preliminary analyses and group characteristics

In total, an average of 7.73% of the trials was deleted due to incorrect identification of the central face. Participants were excluded from further analysis if the number of deleted trials for any of the different trial types was more than 50% (Grol and De Raedt, 2014). This resulted in excluding five participants, two from the vulnerable group and three from the resilient group.

Descriptive statistics for all study variables and task performance for both the vulnerable and the resilient group of the final sample are presented in Table 1. Bivariate correlations among the study variables and ABI scores are presented in Table 2. Apart from the non-significant correlations among FEEL-KJ-MAL and the other self-reported measures, all variables were associated in the theoretically hypothesized direction. Interestingly, PE was positively and significantly correlated with posABI scores, indicating that higher levels of PE were associated with a more broadened attentional breadth for positive emotional stimuli. Preliminary analyses revealed no significant differences in gender, age, FEEL-KJ-MAL, posABI, neuABI, and negABI between the vulnerable and the resilient group, all *p*s > 0.154. Only FEEL-KJ-AD significantly differed between groups, *t* (64) = −4.29, *p* < 0.001, *d* = 0.11, indicating that adaptive ER scores were significantly higher in the resilient group.

**Table 1:** Descriptive statistics.

	Vulnerable (*n* = 22)		Resilient (*n* = 44)	
	M (SD)	min–max	M (SD)	min–max
Gender	27% boys		43% boys	
Age	13.77 (1.90)	11–17	14.41 (1.88)	11–18
PANAS-NE	44.18 (6.05)	38–60	23.51 (3.56)	16–28
PANAS-PE	36.37 (4.78)	25–43	58.38 (6.26)	50–75
FEEL-KJ-AD	119.82 (33.63)	59–195	152.64 (26.91)	105–210
FEEL-KJ-MAL	73.82 (16.66)	51–109	68.89 (23.78)	33–129
P-POS-close	0.74 (0.21)	0.08–1.00	0.70 (0.28)	0.13–1.00
P-POS-far	0.40 (0.21)	0.13–0.78	0.44 (0.23)	0.04–0.96
P-NEU-close	0.70 (0.23)	0.14–1.00	0.68 (0.30)	0.08–1.00
P-NEU-far	0.43 (0.17)	0.17–0.79	0.42 (0.24)	0.07–0.83
P-NEG-close	0.73 (0.24)	0.14–1.00	0.68 (0.28)	0.09–1.00
P-NEG-far	0.42 (0.20)	0.08–0.78	0.41 (0.24)	0.04–0.88

PANAS-NE, negative emotionality; PANAS-PE, positive emotionality; FEEL-KJ-AD, self-reported adaptive emotion regulation; FEEL-KJ-MAL, self-reported maladaptive emotion regulation; P, proportion of correctly localized peripheral targets presented far or close when the central stimulus was positive (POS), neutral (NEU), or negative (NEG).

**Table 2:** Bivariate correlations in the final sample.

	PANAS-NE	PANAS-PE	FEEL-KJ-AD	FEEL-KJ-MAL	posABI	neuABI
PANAS-PE	−0.720**					
FEEL-KJ-AD	−0.590**	0.499**				
FEEL-KJ-MAL	0.018	0.238	−0.148			
posABI	−0.132	0.259**	−0.022	0.120		
neuABI	−0.067	0.189	0.111	0.040	0.693**	
negABI	−0.096	0.146	−0.012	0.240	0.698**	0.657**

PANAS-NE, negative emotionality; PANAS-PE, positive emotionality; FEEL-KJ-AD, self-reported adaptive emotion regulation; FEEL-KJ-MAL, self-reported maladaptive emotion regulation; ABI, attentional broadening index for positive (posABI), neutral (neuABI), and negative (negABI) stimuli. **p < 0.01.

Next, we performed a non-parametric Mann-Withney Test to check for the group differences in the number of deleted trails per trial type as the data on the percentage of deleted trials was non-normally distributed. This test revealed no significant differences between the two groups in terms of the percentage of trials that were deleted for any of the trial types (positive, neutral, negative), all *p*s > 0.497. Non-parametric Friedman’s ANOVA conducted in the total sample revealed that there were no significant differences in the proportion of correctly localized peripheral targets depending on the emotional valence of the presented stimulus if the target stimulus was presented close to the central stimulus, $\chi^{2}$ (2, *N* = 66) = 5.57, *p* = 0.062. However, when the target stimulus was presented far from the central stimulus, significant differences occurred in the proportion of correctly localized peripheral targets depending on the emotional valence, $\chi^{2}$ (2, *N* = 66) = 17.10, *p* < 0.001. Follow-up Wilcoxon’s signed-rank tests show that participants made less errors when identifying neutral compared to positive emotional stimuli (*Z* = −2.68, *p* = 0.007), as well as neutral versus negative emotional stimuli (*Z* = −3.57, *p* < 0.001) when the target stimulus was presented far from the central stimulus.

The 3 Valence (Positive vs. Neutral vs. Negative) x 2 Distance (Far vs. Close) mixed ANOVA on the proportion of correctly localized targets yielded the predicted main effect of distance, *F* (1, 65) = 185.31, *p* < 0.001, $\eta_{p}^{2}\;\;$ = 0.740, but not valence, *F* (2, 130) = 1.05, *p* = 0.353, $\eta_{p}^{2}\;$ = 0.016. This indicates that the proportion of correct identifications of the target stimulus was higher when the target stimulus appeared close compared to far, regardless of the valence of the central stimulus. No significant 3 Valence (Positive vs. Neutral vs. Negative) x 2 Distance (Far vs. Close) interaction effect was found, *F* (2, 130) = 1.36, *p* = 0.260, $\eta_{p}^{2}\;$ = 0.021. Finally, we added mood scores (valence and arousal) at T2 as a covariate to the model in order to check whether concurrent mood significantly affected the overall task performance. No significant interactions with mood at T2 were found, all *p*s > 0.273.

### 3.3. Visual attentional breadth and temperamental differences

**Figure 3:**
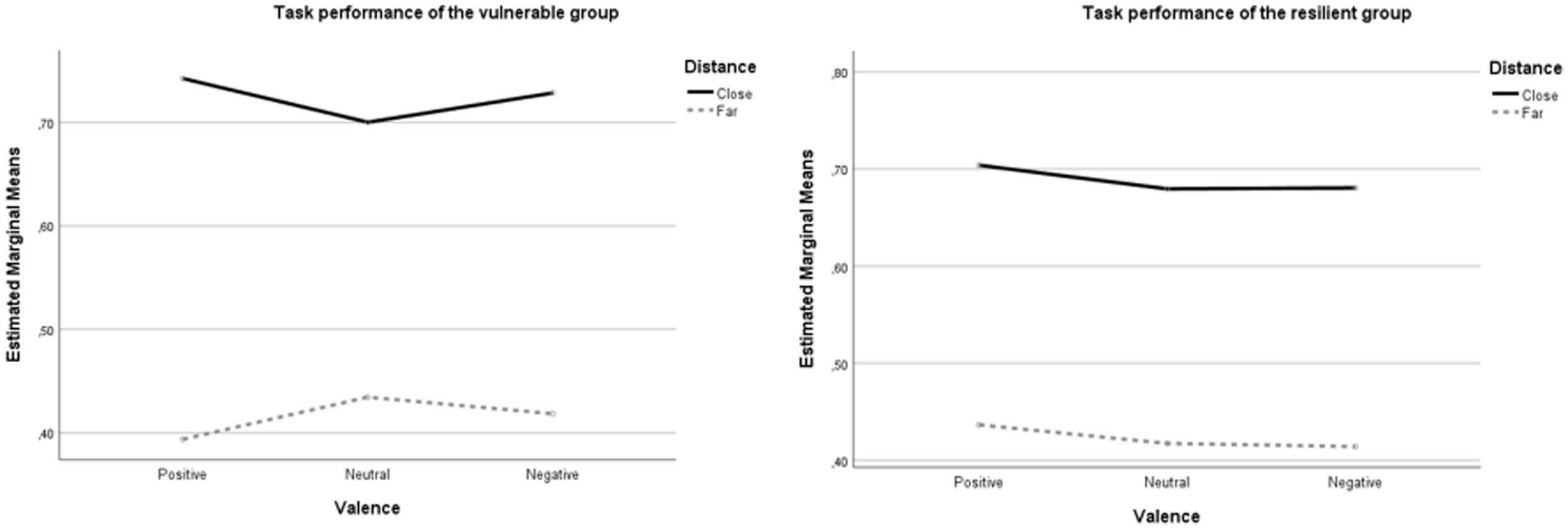
Task performance for the vulnerable and resilient group.

Adding group as a between-subject factor revealed an insignificant 3 Valence (Positive vs. Neutral vs. Negative) × 2 Distance (Far vs. Close) × 2 Group (Vulnerable vs. Resilient) interaction, *F* (2, 128) = 1.21, *p* = 0.30, $\eta_{p}^{2}$ = 0.02. However, after adding the self-reported ER variables in the model as covariates, a significant interaction effect was found between Group, Valence and Distance, *F* (2, 124) = 3.47, *p* = 0.034, $\eta_{p}^{2}$ = 0.053 (Figure 3). Follow-up *between*-group analyses (independent samples *t*-tests) revealed no significant differences in ABI scores (all *p*s > 0.11). However, follow-up *within*-group analyses (paired samples *t*-tests) indicated, that in the vulnerable group, posABI scores (*M* = −0.35, *SD* = 0.23) were significantly higher than neuABI scores (*M* = −0.27, *SD* = 0.21; *t* (21) = 2.53, *p* = 0.020) i.e., vulnerable adolescents showed less attentional broadening toward positive emotional stimuli compared to neutral stimuli. No other *within*-group differences were found, all *p*s > 0.261.

### 3.4. Visual attentional breadth and emotion regulation

The unique effects pertaining to the self-reported ER variables (FEEL-KJ-AD, FEEL-KJ-MAL) in the 3 Valence (Positive vs. Neutral vs. Negative) × 2 Distance (Far vs. Close) × 2 Group (Vulnerable vs. Resilient) ANOVA (in which the self-reported ER variables were added as covariates) revealed no significant interaction effects for FEEL-KJ-MAL (all *p*s > 0.379). However, a significant 3 Valence (Positive vs. Neutral vs. Negative) x 2 Distance (Far vs. Close) x FEEL-KJ-AD interaction emerged, *F* (2, 124) = 3.30, *p* = 0.040, $\eta_{p}^{2}$ = 0.051. Follow-up analyses revealed no significant partial correlations (controlling for temperament group) between ABI scores and overall FEEL-KJ-AD scores. However, post-hoc comparisons between partial ABI – FEEL-KJ-AD correlations revealed a significant difference between the posABI – FEEL-KJ-AD (*r* = −0.136; *Z* = 2.70, *p* = 0.007) and neuABI – FEEL-KJ-AD (*r* = 0.122) correlation, indicating that – albeit non-significant themselves – the posABI – FEEL-KJ-AD and neuABI – FEEL-KJ-AD correlations were significantly different from one another. More specifically, attentional *narrowing* toward positive emotional stimuli was related to more adaptive ER whereas, for neutral stimuli, attentional *broadening* toward such information was related to more adaptive ER. No other differences were found, all *p*s > 0.05.

### 3.5. Temperament, visual attentional breadth, and emotion regulation

Results pertaining to the hypothesized multiple mediation models revealed that none of the indirect effects were significant (see Table 3).

**Table 3:** Indirect effects of group on emotion regulation through attentional broadening (multiple mediation results).

IV: Group	DV: FEEL-KJ-AD			DV: FEEL-KJ-MAL		
	*B*	SE	CI	*B*	SE	CI
M: posABI	−5.05	4.44	[−15.45, 2.13]	2.69	3.20	[−2.75, 10.03]
M: neuABI	0.38	4.11	[−8.79, 8.24]	0.21	1.55	[−2.20, 4.18]
M: negABI	−0.87	2.00	[−5.23, 3.19]	−0.20	2.05	[−4.74, 4.26]

IV, independent variable; DV, dependent variable; M, mediator; FEEL-KJ-AD, self-reported adaptive emotion regulation; FEEL-KJ-MAL, self-reported maladaptive emotion regulation; ABI, attentional broadening index for positive (posABI), neutral (neuABI), and negative (negABI) stimuli.

### 3.6. Post-hoc analyses

Given the lack of findings for the hypothesized mediation models, we ran six additional simple mediation models in which a single mediator was tested (i.e., posABI, neuABI, and negABI). Three models included FEEL-KJ-AD as the DV, and three models included FEEL-KJ-MAL as the DV. None of the indirect effects were significant (see Table 4).

**Table 4:** Indirect effects of group on emotion regulation through attentional broadening (simple mediation results).

IV: Group	DV: FEEL-KJ-AD			DV: FEEL-KJ-MAL		
	*B*	SE	CI	*B*	SE	CI
M: posABI	−3.83	2.91	[−10.54, 0.90]	2.43	2.49	[−2.39, 7.63]
M: neuABI	−3.81	3.33	[−11.79, 1.17]	0.92	2.28	[−3.34, 5.99]
M: negABI	−0.10	1.35	[−3.00, 2.84]	−0.07	1.21	[−2.46, 2.96]

IV, independent variable; DV, dependent variable; M, mediator; FEEL-KJ-AD, self-reported adaptive emotion regulation; FEEL-KJ-MAL, self-reported maladaptive emotion regulation; ABI, attentional broadening index for positive (posABI), neutral (neuABI), and negative (negABI) stimuli.

Furthermore, we also performed additional post-hoc analyses in order to test the reversed relationships between ER strategies and visual attentional breadth. The decision to further test the temperament – ER strategies – visual attentional breadth relationship was based on the findings of a previous study on the attentional scope model of rumination in college students (Grol et al., 2015) suggesting that the reversed relationship may just be as likely, i.e., that the ER strategies that are shaped by one’s temperament may, for their part, also affect visual attentional breadth.

**Figure 4:**
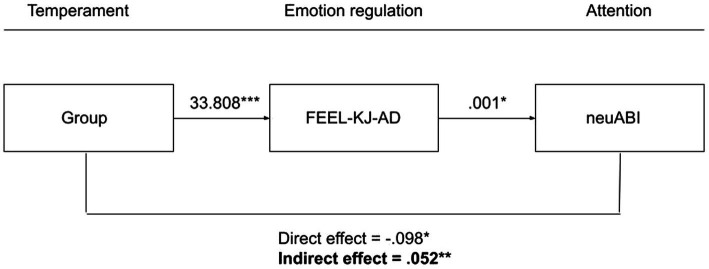
Post-hoc mediation model including the reversed relationship between adaptive emotion regulation strategies (FEEL-KJ-AD) and visual attentional breadth for neutral stimuli (neuABI).

Post-hoc analyses revealed that the indirect effect of group on neuABI through FEEL-KJ-AD was significant (Table 5; Figure 4). Although the direct effect of temperament group on neuABI suggested otherwise, results pertaining to this mediation model indicated that temperamental resilient individuals will show more adaptive ER strategies which in turn will lead to more attentional broadening toward neutral stimuli in these youth. All other indirect effects were non-significant (see Table 5).

**Table 5:** Indirect effects of the post-hoc analyses.

M: FEEL-KJ-AD									
IV: Group	DV: posABI			DV: neuABI			DV: negABI		
	B	SE	CI	B	SE	CI	B	SE	CI
	−0.03	0.02	[−0.07, 0.00]	**0.05**	**0.02**	**[0.01, 0.09]**	−0.01	0.02	[−0.06, 0.03]
M: FEEL-KJ-MAL									
IV: Group	DV: posABI			DV: neuABI			DV: negABI		
	B	SE	CI	B	SE	CI	B	SE	CI
	−0.00	0.01	[−0.02, 0.01]	0.00	0.01	[−0.01, 0.02]	0.00	0.01	[−0.02, 0.01]

IV, independent variable; DV, dependent variable; M, mediator; FEEL-KJ-AD, self-reported adaptive emotion regulation; FEEL-KJ-MAL, self-reported maladaptive emotion regulation; ABI, attentional broadening index for positive (posABI), neutral (neuABI), and negative (negABI) stimuli. Bold values significant indirect effect.

## 4. Discussion

The current study explored the role of visual attentional breadth for emotional (i.e., positive, neutral, and negative) information in the relationship between temperament and ER strategies in adolescents using a previously validated performance-based measure of visuospatial attentional breadth (Bosmans et al., 2009; Grol and De Raedt, 2014; Grol et al., 2015). Overall, the current study had three main findings pertaining to our aims. First, PE was positively associated with attentional broadening for positive emotional stimuli and temperamental vulnerable adolescents showed particular deficits in the processing of positive emotional stimuli when this information was presented far. Second, visual attentional breadth toward neutral stimuli was positively related to adaptive ER strategies. Third, no evidence was found for the proposed mediation models. However, post-hoc analyses provided preliminary evidence for a reversed mediation model in which adaptive ER strategies mediated the relationship between temperament and visual attentional breadth toward neutral stimuli. The results underscore the apparent complexity of the relations between individual factors and individual differences in visual attentional breadth and point out the need for further research disentangling the role of both positive and negative emotions, as well as related resilience and vulnerability factors, in adolescents.

The first aim sought to examine the relationship between temperament and visual attentional breadth and to investigate whether the emotional valence of the stimuli in the target of attention would play a role in this relationship. Drawing from the broaden-and-build theory of positive emotions and prior work in adults (Fredrickson, 1998, 2001; Fredrickson and Branigan, 2005), we hypothesized that temperamental vulnerable adolescents would show a more narrowed attentional focus, especially toward negative and positive emotional stimuli, compared to temperamental resilient adolescents. First, bivariate correlations revealed a significant and positive correlation between PE and visual attentional breadth toward positive emotional stimuli. Second, adolescents from the temperamental vulnerable group showed a hampered visual attentional breadth toward positive emotional stimuli when presented far as compared to neutral stimuli, which – in the light of the aforementioned finding – may be attributed to their low levels of PE. Albeit preliminary, these results suggest that individual factors, such as one’s biological disposition to experience positive emotions, should also be considered in future work on visual attentional breadth for emotional information as they may contribute to individual differences in the broadening effects of positive emotions as proposed by the broaden-and-build theory (Fredrickson, 1998, 2001; Fredrickson and Branigan, 2005). However, results should be interpreted with caution as no direct evidence for our first hypothesis related to temperament and visual attentional breadth was found. One possible explanation for the lack in clear-cut findings pertaining to our first aim may lay in the methodology used in the current study, i.e., all participants watched a 3–min film clip prior to the task in order to guarantee a neutral baseline. Given that temperament reflects reactivity toward negative (vulnerable group) or positive (resilient group) emotions, it may be that the temperamental constellation of the temperament groups was not triggered in the current study. Perhaps, the relationship between temperament and visual attentional breadth may become more apparent after a negative or positive mood induction (Grol and De Raedt, 2014; Grol et al., 2015).

The second aim sought to examine the unique relationships between ER strategies and visual attentional breadth and to investigate whether the valence of the stimuli presented in the target of attention would play a role in this relationship. Based on the broaden-and-build theory (Fredrickson, 2001) and the attentional scope model of rumination (for review see Whitmer and Gotlib, 2013) we hypothesized a more broadened attentional focus, especially for positive emotional stimuli, to indicate a greater reliance on adaptive ER strategies, whereas a more narrowed attentional focus, especially for negative emotional stimuli, was hypothesized to be positively related to greater maladaptive ER strategies. When investigating the relations among visual attentional breadth and ER strategies, a significant interaction emerged between visual attentional breadth and adaptive ER strategies. Follow-up analyses indicated that correlations between indicators of visual attentional breadth and adaptive ER strategies were significantly different for positive versus neutral stimuli, indicating that higher adaptive ER strategies were associated with attentional narrowing for positive emotional stimuli and attentional broadening for neutral stimuli. The latter finding was also replicated in the post-hoc mediation analyses. First, this finding lays in line with previous results in adults (Isen, 2000; Wadlinger and Isaacowitz, 2006) that visual attentional breadth for neutral information is related to indicators of resilience such as adaptive ER strategies. Second, research has shown that *distraction* – an adaptive ER strategy – requires individuals to replace existing emotional information with independent neutral information and *reappraisal* – also an adaptive ER strategy – to modify existing and incoming emotional information *via* neutral reinterpretation (Sheppes and Gross, 2011). The ability to consider neutral information may thus serve as an indicator of a healthy adaptive ER strategy use a first explanation for our finding that adaptive ER strategies were not significantly related to visual attentional breadth for positive emotional stimuli can be found in the Hedonic Contingency Model (HCM; Wegener and Petty, 1994). The HCM posits that resilient individuals are conditioned through experience to intentionally pursue activities that will prolong or elevate their benevolent affective state. The HCM thus suggest that resilient individuals will broaden their attention in response to stimuli that are of similar valence as their current affective state in order to maintain this state (Wegener and Petty, 1994; Wadlinger and Isaacowitz, 2006), which can be considered an adaptive way of regulating one’s emotions. Given that we chose to implement a neutral baseline in the current study, it may be that individuals with average adaptive ER abilities were more prone to neutral stimuli in our study with the aim of sustaining their neutral mood. A second explanation for the finding that adaptive ER strategies did not significantly predict visual attentional breadth for positive information can be found in more recent theoretical models of ER. These models suggest that the classification of ER strategies as adaptive or maladaptive is too simplistic. Instead adaptive ER implies the flexible use of a range of ER strategies to meet contextual demands (Blanke et al., 2020). In line with this, previous work in adolescents has revealed that high trait levels of *distraction* resulted in more attentional narrowing (Boelens et al., 2021). Distraction is only seen as an adaptive strategy when it is flexibly used and combined with other strategies such as acceptance (Wolgast and Lundh, 2017). A high score on trait adaptive ER as measured in the current study might thus not fully capture true adaptive ER strategy usage.

Our third and final aim was to test the proposed mediation model in which visual attentional breadth was expected to mediate the relation between temperament and ER strategies. More specifically, we hypothesized the relationship between temperamental resiliency and adaptive ER strategies to be mediated by attentional broadening, especially toward positive and neutral emotional stimuli and the temperamental vulnerability – maladaptive ER strategies relationship to be mediated by attentional narrowing, especially toward negative emotional stimuli. Unfortunately, no significant mediation effects emerged. Considering the results pertaining to our first and second aim, the lack of findings may lay in the methodology used in the current study, i.e., the implementation of a neutral baseline, and the use of a self-report questionnaire to study adaptive and maladaptive ER. An avenue for future research will be to test the proposed mediation models after inducing a negative and a positive mood, and by using different methods to capture ER in daily life such as experience sampling methods (Blanke et al., 2020). It is likely that the model pertaining to temperamental resiliency and adaptive ER strategies will become more apparent after a positive mood induction, whereas the same line of reasoning may hold for the temperamental vulnerability – maladaptive ER strategies model under a negative mood induction.

Because of the lack in findings pertaining to the proposed mediation models, we decided to conduct post-hoc analyses based on the findings of a previous study on the attention scope model of rumination in college students (Grol et al., 2015). Principally, it was assumed that trait ER strategies would also affect visual attentional breadth, i.e., that the reversed relationship would (also) hold. For example, in the study of Grol et al. (2015) it was proposed that trait ruminators will show a more narrowed attentional scope in general as they have spent more time at – and elaborated more on – thinking about stressful events in a ruminative manner throughout their lives, which may’ve resulted in an overall narrowed attentional scope over time. A similar reasoning may apply for adaptive ER strategies. Individuals with good adaptive ER abilities may’ve elaborated more on – and spent more time at generating alternative thoughts or behaviors to successfully downregulate negative emotions, which may’ve resulted in a default broadened attentional scope over time. In line with this line of thought, post-hoc analyses revealed that a model in which adaptive ER strategies mediated the relationship between temperament group membership and visual attentional breadth toward neutral stimuli was significant. First, conform the theoretical proposition by integrated cognitive-affective models of depression (e.g., Yap et al., 2007; Hyde et al., 2008) and previous studies in younger age groups (Verstraeten et al., 2009, 2012; Mezulis et al., 2011; Yap et al., 2011; Mezulis and Rudolph, 2012; Harding et al., 2014; Hudson et al., 2015; Van Beveren et al., 2017, 2019a), temperamental resilient adolescents reported significantly more adaptive ER strategies than their vulnerable peers. Second, conform our hypothesis, adaptive ER strategies were – in turn – significantly and positively associated with visual attentional breadth toward neutral stimuli, indicating that adolescents high in adaptive ER strategies tend to show more visual attentional breadth in the context of neutral information.

Overall, the finding that this alternative – partially reversed – mediation model was significant is partly in line with the reasoning of the attentional scope model of rumination (Whitmer and Gotlib, 2013) and the findings of a previous study in college students (Grol et al., 2015) that trait ER strategies also affect visual attentional breadth. Our findings add to this literature by showing that this may not only be the case for maladaptive ER strategies such as rumination, but also for adaptive ER strategies. More specific, our findings suggest that temperamental resilient adolescents will show more adaptive ER strategies because of their temperamental constellation which – in turn – results in a more broadened attentional breadth for neutral information. However, our findings do not rule out the possibility that the relationship between ER strategies and visual attentional breadth is bidirectional. It seems for example plausible that the originally proposed model may particularly hold for understanding the development of one’s preferred ER-repertoire from early-childhood to adolescence (Eisenberg et al., 2010; Gross, 2013) i.e., when ER strategies occur and how they develop, whereas the partially reversed mediation model may explain the self-perpetuating nature and thus conservation of these ER strategies, i.e., how ER strategies become more ‘trait-like’. Clearly, future efforts for testing the directions of causality among temperament, ER strategies, and visual attentional breadth are warranted to confirm these plausible presumptions.

### 4.1. Limitations

The current study was the first to investigate the interrelations between temperament, visual attentional breadth for emotional information, and emotion regulation strategies in a selected sample of adolescents. Despite some notable strengths, some limitations deserve mention. First, hypotheses were tested using a selected-sample of adolescents. Given that visual attentional breadth for emotional information may be particularly disturbed in individuals meeting the criteria for clinical MDD, future replication efforts may utilize clinical samples. Also, due to our sampling approach we were unable to disentangle the unique effects of each of the temperament dimensions. In the future, one may wish to implement a dimensional approach for operationalizing the NE and PE temperament dimensions. Third, the neutral mood-induction may not have triggered participants’ temperamental vulnerability/resilience. Future studies testing the proposed models under a negative/positive induced mood is warranted. Fourth, results should be interpreted with caution due to the relatively small sample size. Future research may wish to replicate the current study’s findings in a larger sample. Fifth, more ecologically valid methods should be considered to capture attentional processes in real-life settings such as virtual reality glasses. Sixth, a flexible view of ER, rather than the simplistic categorization of ER strategies in adaptive or maladaptive strategies, can be used to investigate the specific relationship between temperament, attentional breadth and ER (e.g., by using experience sampling methods; Blanke et al., 2020). Finally, future longitudinal studies with multiple points in time are warranted for testing the causality and directionality of the relationships among temperament, ER strategies, and visual attentional breadth for emotional information.

In conclusion, the current study is novel in investigating the associations between temperament, visual attentional breadth for emotional information, and emotion regulation strategies in adolescents. Overall, our aim was to investigate the role of visual attentional breadth for emotional information in the distinct pathways from temperament to emotion regulation strategies in a selected sample of adolescents using a previously validated performance-based measure of visuospatial attentional breadth. Although no direct evidence was found for the proposed mediational pathways, our results extend the broaden-and-build theory (Fredrickson, 1998, 2001; Fredrickson and Branigan, 2005) by showing that the broadening effect of positive emotions may be dependent on stable child characteristics. In addition, our results suggest that a broadened attentional scope toward neutral information may be an indicator of one’s adaptive ER abilities. Lastly, results suggest a reversed form of the proposed mediation model that could explain how emotion regulation strategies become more trait-like. These findings underscore the apparent complexity of the relations between individual factors and individual differences in visual attentional breadth and point out the need for further prospective research disentangling the interactive relations between individual characteristics, momentary mood, and visual attention in both clinical and larger selected sample of adolescents.

## Data availability statement

The raw data supporting the conclusions of this article will be made available by the authors, without undue reservation.

## Ethics statement

The studies involving human participants were reviewed and approved by Ethical Committee of the Faculty of Psychology and Educational Sciences at Ghent University (Belgium). Written informed consent to participate in this study was provided by the participants’ legal guardian/next of kin.

## Author contributions

M-LB: conceptualization, introduction, methodology, data analyses, and writing–original draft preparation. JB: administration, revision manuscript, methodology, data analyses. RR: methodology, data-analyses, review, and editing. MG: methodology, data-analyses, review, and editing. LW: methodology, review, and editing. CB: conceptualization, methodology, review and editing, and supervision. All authors contributed to the article and approved the submitted version.

## Conflict of interest

The authors declare that the research was conducted in the absence of any commercial or financial relationships that could be construed as a potential conflict of interest.

## Publisher’s note

All claims expressed in this article are solely those of the authors and do not necessarily represent those of their affiliated organizations, or those of the publisher, the editors and the reviewers. Any product that may be evaluated in this article, or claim that may be made by its manufacturer, is not guaranteed or endorsed by the publisher.

## Footnotes

^1^ http://kt.ijs.si/data/Emoji_sentiment_ranking/
